# Gut microbiota-driven regulation of queen bee ovarian metabolism

**DOI:** 10.1128/spectrum.02145-23

**Published:** 2023-09-26

**Authors:** Wan-Li Li, Qi Huang, Jia-Li Li, Ping Wu, Bangrong Wei, Xi-Jie Li, Qi-He Tang, Zhi-Xiang Dong, Jian Xiong, Hong Tang, Jun Zhang, Chong-Hui Zhao, Zhe Cao, Yuan Chen, Wen-zheng Zhao, Kai Wang, Jun Guo

**Affiliations:** 1 Faculty of Life Science and Technology, Kunming University of Science and Technology, Kunming, Yunnan province, China; 2 Nanchuan District Livestock, Veterinary and Fisheries Center, Chongqing, China; 3 Chongqing Nanchuan District Livestock, Veterinary and Fishery Center, Chongqing, China; 4 Yunnan Zhongfeng Technology Development Co. Ltd., Kunming, Yunnan, China; 5 Chongqing Nanchuan Bee Breeding Center, Chongqing, China; 6 Pujia Life Technology (Fuzhou) Co., LTD, Fuzhou, China; 7 Faculty of Animal Science and Technology, Yunnan Agricultural University, Heilongtan, North Suburb, China; 8 State Key Laboratory of Resource Insects, Institute of Apicultural Research, Chinese Academy of Agricultural Sciences, Beijing, China; South China Sea Institute of Oceanology, Chinese Academy of Sciences, Guangzhou, Guangdong, China

**Keywords:** gut microbiota, ovary, queen bee, oviposition restriction, metabolomics

## Abstract

With the global prevalence of *Varroa* mites, more and more beekeepers resort to confining the queen bee in a queen cage to control mite infestation or to breed superior and robust queen bees. However, the impact of such practices on the queen bee remains largely unknown. Therefore, we subjected the queen bees to a 21-day egg-laying restriction treatment (from the egg stage to the emergence of adult worker bees) and analyzed the queen bees’ ovarian metabolites and gut microbiota after 21 days, aiming to assess the queen bees’ quality and assist beekeepers in better hive management. Our findings revealed a significant reduction in the relative expression levels of *Vg* and *Hex110* genes in the ovaries of egg laying-restricted queen bees compared to unrestricted egg-laying queens. The diversity of gut microbiota in the queen bee exhibited a notable decrease, accompanied by corresponding changes in the core bacteria of the microbial community, the relative abundance of *Lactobacillus* and *Bifidobacterium* increased from 22.34% to 53.14% (*P* = 0.01) and from 0.053% to 0.580% (*P* = 0.04), respectively. The relative abundance of *Bombella* decreased from 25.85% to 1.720% (*P* = 0.002). Following egg-laying restriction, the activity of the queen bee’s ovaries decreased, while the metabolism of glycerophospholipids remained or stored more lipid molecules, awaiting environmental changes for the queen bee to resume egg laying promptly. Furthermore, we observed that *Bombella* in the queen bee’s gut may regulate the queen’s ovarian metabolism through tryptophan metabolism. These findings provide novel insights into the interplay among queen egg laying, gut microbiota, and ovarian metabolism.

**IMPORTANCE:**

With *Varroa* mite infestation, beekeepers often confine the queen bee in cages for control or breeding. However, the impact on the queen bee is largely unknown. We evaluated queen bee quality by restricting egg laying and analyzing ovarian metabolites and gut microbiota. In this study, we provided a comprehensive explanation of the expression of ovarian genes, the diversity of gut microbiota, and changes in ovarian metabolism in the queen bee. Through integrated analysis of the queen bee’s gut microbiota and ovarian metabolism, we discovered that the gut microbiota can regulate the queen bee’s ovarian metabolism. These findings provide valuable insights into the interplay among egg laying, gut microbiota, and the reproductive health of the queen bee. Understanding these relationships can contribute to the development of better strategies for *Varroa* mite control and queen bee breeding.

## INTRODUCTION

Honeybees are highly social insects. A honeybee colony usually consists of a queen, tens of thousands of worker bees, and a few males who are interdependent and work together to form an organic whole (1). Honeybees are also one of the most important pollinators, pollinating 87.5% of angiosperms. A honeybee visits approximately 2,000 flowers per day while foraging for nectar and pollen (2). In addition, honeybees produce valuable products such as honey, propolis, pollen, beeswax, and royal jelly.

However, honeybee colonies have declined dramatically worldwide in recent years (3), and the factors contributing to this situation are multiple and complex. Pathogens, bacteria, and fungi can cause colony collapse disorder (4 – 6). Additionally, the widespread use of pesticides and herbicides, as well as the prevalence of parasites such as *Varroa* mites, can all contribute to the decline of honeybee populations (7 – 9). Moreover, viruses are one of the major threats to honeybee colony decline (10).

In addition to the above factors, queen failure and loss are also considered major causes of colony decline (11, 12). The queen is the central part of the colony and plays a vital role. The queen regulates the colony’s population and potential by releasing pheromones and laying eggs (13), and these pheromones also inhibit worker bees from laying eggs (14, 15). Queen fertility is a critical factor for the colony, and colony productivity is most directly related to the overall reproductive health of the queen. A healthy queen that is in the best egg-laying period is crucial for the existence of the colony (16). This is not only because the queen can lay many high-quality eggs but also because a young and strong queen has the most control over the colony. Therefore, beekeepers usually use young, strong queens to manage the colony for better beekeeping (17).

However, if a serious disease occurs in the colony or the number of *Varroa* mites threatens the colony’s health, the colony will soon collapse if the situation is not controlled. Nevertheless, the use of acaricides in beekeeping should be minimized to avoid chemical residues that can accumulate in honey and beeswax (18). Today, in order to eliminate diseases and control *Varroa* mite populations, beekeepers typically confine the queen in a cage for 21 days (from the egg stage to the emergence of adult workers). They also use oxalic acid, a natural, non-toxic organic acid, as an acaricide to control the mite population in the colony and prevent the queen from laying eggs or spreading viruses in the colony (19 – 21).

Meanwhile, restricting queen egg production significantly increases egg weight and size; egg weight and size have a significant effect on the weight, thoracic length, and thoracic width of the queen (22). The expression level of the vitellogenin (*Vg*) gene in the queen’s abdomen also significantly increases with increasing restriction time. However, the expression level of the vitellogenin receptor (*Vgr*) in the ovary remains constant to provide vitellogenin for oviposition immediately after environmental improvement (23). The use of oviposition restriction to improve queen laying productivity has been widely used in production practices. In the early years of nectar abundance, beekeepers use queen confinement to obtain a large number of foragers and thus obtain high yields. The use of oviposition restriction to produce a large number of overwintering bees is also one of the important measures for honey bee management in all seasons (24). Recent research has shown that using oviposition restriction to treat queen bees results in larger eggs, which in turn produce high-quality queens (25).

In addition to the above types of artificial restrictions on queen egg production, the queen bees will also reduce egg production on their own. As the optimum temperature range for bees to live is between 15 and 25℃ (26), bees can come out of the hive, the queen can lay eggs, and worker bees can nurse larvae when the temperature is between 5 and 35℃ (27). This period can be called the breeding period and is also the time for producing various bee products. When the temperature is below 10°C for a long time, the queen stops laying eggs; the bees reduce their activities outside the hive and form clusters inside the hive; and the colony enters the overwintering period (28).

As the colony enters the reproductive period, some queens may leave the hive to find a new home due to environmental changes and lack of food. During this period, the worker bees will feed the queen less frequently (29), or the queen may actively refuse to be fed by the worker bees; the queen will lose weight; the queen will reduce the number of eggs she lays; and egg production will drop dramatically (30). Therefore, the queen can easily fly and take the worker bees out of the hive. Certainly, beekeepers also refer to this phenomenon as “swarming.”

Many beekeepers also sell queens, which are placed in a small cage and escorted safely to their destination by a few worker bees (31). Upon arrival, the queen often becomes depressed and does not lay eggs, and will only try to start laying after a few days of acclimatization in the colony (32). Whether this restriction of queen laying or reduction in queen laying affects the queens themselves has not been studied.

In this study, we placed the queen bee in a queen cage and restricted the queen bee from laying eggs and left the queen bee’s ovaries in a closed state. To investigate whether the queen ovaries change and whether this change affects the queen ovarian development and egg production, we used metabolomics to identify pathways and differential metabolites that were significantly affected in ovaries after spawning restriction. Moreover, we will also analyze this change in terms of gut bacteria. Previous studies have shown that the diversity of the queen’s gut flora is relatively homogeneous compared to that of the worker bees, and that the queen’s gut microbiota changes, depending on the environment (33). Therefore, we analyzed the gut microbiota of queen bees and found that the gut microbiota of queen bees changed after oviposition restriction; the richness of the gut microbiota of queen bees decreased (34); and the two groups were significantly separated. In addition, we determined the expression of important genes in the ovaries after the queen was restricted to laying eggs. These differences and variations help us to investigate whether the queen’s ovaries, when in a closed state, affect the queen and thus the development of the whole colony, causing losses to the colony and the beekeeper.

## MATERIALS AND METHODS

### Sample collection

This experiment was conducted in the apiary of Kunming University of Science and Technology in May 2022. Twenty healthy, disease-free colonies of honey bees (*Apis mellifera*) were selected, and their colony strength, sealed brood area, and food supply were equal. The queen of each colony was a mated queen of the same species and age, healthy, and in egg-laying condition; the age of all 20 queen bees is 1 year. Ten colonies were randomly selected as one group, and each queen was placed in the queen cage (CQ), while the other groups of 10 colonies were left with the queens untreated (FQ). After 21 days of captivity (35), all 20 queens were removed from the colonies and stored at −80°C for subsequent experiments.

### Dissection of the queen bee intestine and ovary

All experiments are performed in the ultra-clean table. First, take the queen bee out of the −80°C refrigerator and place it on ice to thaw. After thawing, soak the queen bee sample in 75% alcohol for two minutes, then place the queen bee sample in phosphate-buffered saline (PBS) for cleaning. Then, a pair of forceps was used to hold the queen bee’s body in place. Scissors were used to lift the scales off the queen bee’s abdomen starting from the caudal end of the stomach and along the sides of the abdomen. At that point, we could see the ovaries and intestinal tissues in the abdominal cavity of the queen bee. As described in Prešern and Smodiš Škerl (36), the scales of the queen bee’s abdomen were then fixed with a needle and the ovaries and entire intestinal tissues were removed with another pair of forceps but excluding the honey crop. Finally, the intestinal and ovarian tissues were snap frozen with liquid nitrogen and stored at −80°C.

### Intestinal DNA extraction and sequencing

The dissected queen bee gut was transferred to a 1.5-mL microcentrifuge tube containing 100 µL of double-distilled water and ceramic beads (0.1 mm) for subsequent DNA extraction.

Queen bee gut samples were homogenized in a tissue lyser and the lysed samples were then subjected to DNA extraction using the Insect DNA Kit (Do926-02; Omega, Inc., USA). Total DNA was eluted in 50-µL elution buffer according to the manufacturer’s instructions, and the quality of the extracted DNA was assessed using NanoDrop 2000 (Thermo Scientific, Wilmington, USA) and 2% agarose gel electrophoresis to measure and evaluate the concentration and quality of the extracted DNA.

The hypervariable region V3-V4 of the bacterial 16S rRNA gene was amplified with primer pairs 338F (5′-ACTCCTACGGGAGGCAGCAG-3′) and 806R (5′-GGACTACHVGGGTWTCTAAT-3′) (37) by T100 Thermal Cycler PCR thermocycler (Bio-Rad, USA). The PCR reaction mixture including 4-µL 5× Fast Pfu buffer, 2-µL 2.5-mM deoxy-ribonucleoside triphosphate (dNTP), 0.8 µL of each primer (5 µM), 0.4-µL Fast Pfu polymerase, 10 ng of template DNA, and ddH_2_O to a final volume of 20 µL. PCR amplification cycling conditions were as follows: initial denaturation at 95℃ for 3 min, followed by 27 cycles of denaturing at 95℃ for 30 s, annealing at 55℃ for 30 s and extension at 72℃ for 45 s, single extension at 72℃ for 10 min, and end at 4℃. The PCR product was extracted from 2% agarose gel and purified using the PCR Clean-Up Kit (YuHua, Shanghai, China) according to manufacturer’s instructions and was quantified using Qubit (version 4.0; Thermo Fisher Scientific, USA). Purified amplicons were pooled in equimolar amounts and paired-end sequenced on an Illumina PE300 platform (Illumina, San Diego, USA) according to the standard protocols by Majorbio Bio-Pharm Technology Co. Ltd. (Shanghai, China).

After demultiplexing, the resulting sequences were quality filtered with fastp (0.19.6) and merged with FLASH (version 1.2.11). Then, the high-quality sequences were de-noised using DADA2 (38) plugin in the Qiime2 (version 2020.2) pipeline with recommended parameters, which obtains single-nucleotide resolution based on error profiles within samples. DADA2 de-noised sequences are usually called amplicon sequence variants (ASVs). Remove all sequences annotated as chloroplasts and mitochondria from all samples. To minimize the effects of sequencing depth on alpha and beta diversity measure, the number of sequences from each sample was rarefied to 20,000, which still yielded an average Good’s coverage of 97.90%. The ASV abundance represents the abundance value of each ASV in each sample (i.e., the corresponding count of all sequences). Typically, when analyzing the microbial composition among samples, the ASV or species sequence count in each sample is divided by the total sequence count of the sample to obtain the relative abundance (proportion) of each ASV or species. Taxonomic assignment of ASVs was performed using the naive Bayes consensus taxonomy classifier implemented in Qiime2 and the SILVA 16S rRNA database (version 138).

### Sample preparation for metabolite extraction

Fifty milligrams of the queen ovary sample was weighed into a 2-mL centrifuge tube, and a 6-mm diameter grinding bead was added. Extract (400 µL; methanol:acetonitrile = 1:1, vol/vol) containing four internal standards (L-2-chlorophenylalanine, etc.) was added. Cryogenic tissue grinder was used for 6 min (−10°C, 50 Hz). Ultra-sonic extraction was performed at low temperature for 30 min (5°C, 40 KHz). The samples were placed at −20°C for 30 min, centrifuged for 15 min (13,000 *g*, 4°C), and the supernatant was transferred to an injection vial with an internal cannula for analysis. In addition, 20 µL of supernatant was removed from each sample, mixed, and used as a quality control (QC) sample.

### Untargeted metabolomics profiling of queen bee ovary

The instrumental platform for this LC-MS analysis was an ultra-high-performance liquid chromatography-tandem Fourier transform mass spectrometry UHPLC-Q Exactive HF-X system (Thermo Scientific).

Chromatographic conditions were as follows: the column was an ACQUITY UPLC HSS T3 (100 mm × 2.1 mm i.d., 1.8 µm; Waters Corporation, Milford, USA); mobile phase A was 95% water + 5% acetonitrile (containing 0.1% formic acid); mobile phase B was 47.5% acetonitrile + 47.5% isopropanol + 5% water (containing 0.1% formic acid), and the injection volume was 3 µL. The column temperature was 40°C.

Mass spectrometry conditions were as follows: samples were subjected to electrospray ionization, and mass spectra were acquired in positive and negative ion scanning modes. The scan range was 70–1,050 *m*/*z*; the sheath gas flow rate was 50 arb; the auxiliary gas flow rate was 13 arb; the heating temperature was 425°C; the capillary temperature was 325°C; the spray voltage (+) was 3500 V; the spray voltage (−) was −3500 V; and the S-lens voltage was 50.

We first injected three QC samples to balance the system and column. In the analysis process, one QC sample was injected after every three samples to monitor instrument stability.

### RNA extraction and RT-PCR

Ovary samples were removed from −80°C and placed on ice, and the entire procedure was performed on ice. RNA was extracted from the ovaries of queen bees using Trizol (Invitrogen, Carlsbad, CA, USA), and the concentration of RNA was measured using NonDrop 2000 (Thermo Scientific). The extracted RNA was then reverse transcribed using Takara kit (TaKaRa, Dalian, China). The reverse transcribed cDNA is frozen at −20°C until use.

We selected six genes [*Vgr*, *Vg*, juvenile hormone acid methyltransferase (*Jhamt*), *Hex110*, *Tor*, and *Egfr*] (Table S1) using *Actin* as a reference gene and performed a 10-fold dilution of the cDNA. A total of 1.6-µL forward and reverse primers, 2-µL CDNA solution, 10-µL TB Green Premix Ex Taq, and the addition of sterilized water to 20 µL. Amplification was performed using the following cycling conditions: 95°C for 30 s, 40 cycles of 95°C for 5 s, 60°C for 30 s, then 95°C for 15 s, 60°C for 1 min, and 95°C for 15 s. We used the 2^−ΔΔCT^ method and calculated the mean of three technical replicates (39). Calculation of relative differential gene expression in queen ovaries was performed after limiting queen oviposition.

### Statistical analysis

Independent samples *t*-tests were used to calculate the variability of ovary weight between the two groups of queens using the built-in method of GraphPad Prism version 8.0.2. The chao and sobs indices of alpha diversity were used in the same way.

The raw metabolomic data were imported into the metabolomics software Progenesis QI (Waters Corporation) for baseline filtering, peak identification, integration, retention time correction, and peak alignment, resulting in a data matrix containing retention time, mass-to-charge ratio, and peak intensity information. The MS (mass spectrum) and MS/MS (tandem mass spectrometry) mass spectra were matched to the metabolism database with the MS mass error set to less than 10 ppm, and the metabolites were identified based on the secondary mass spectra matching score. Databases included METLIN (https://metlin.scripps.edu/), the human metabolome database (http://www.hmdb.ca), and Lipid Maps (http://www.lipidmaps.org).

## RESULTS

### Gene expression in queen ovaries and ovary weight

**FIG 1:**
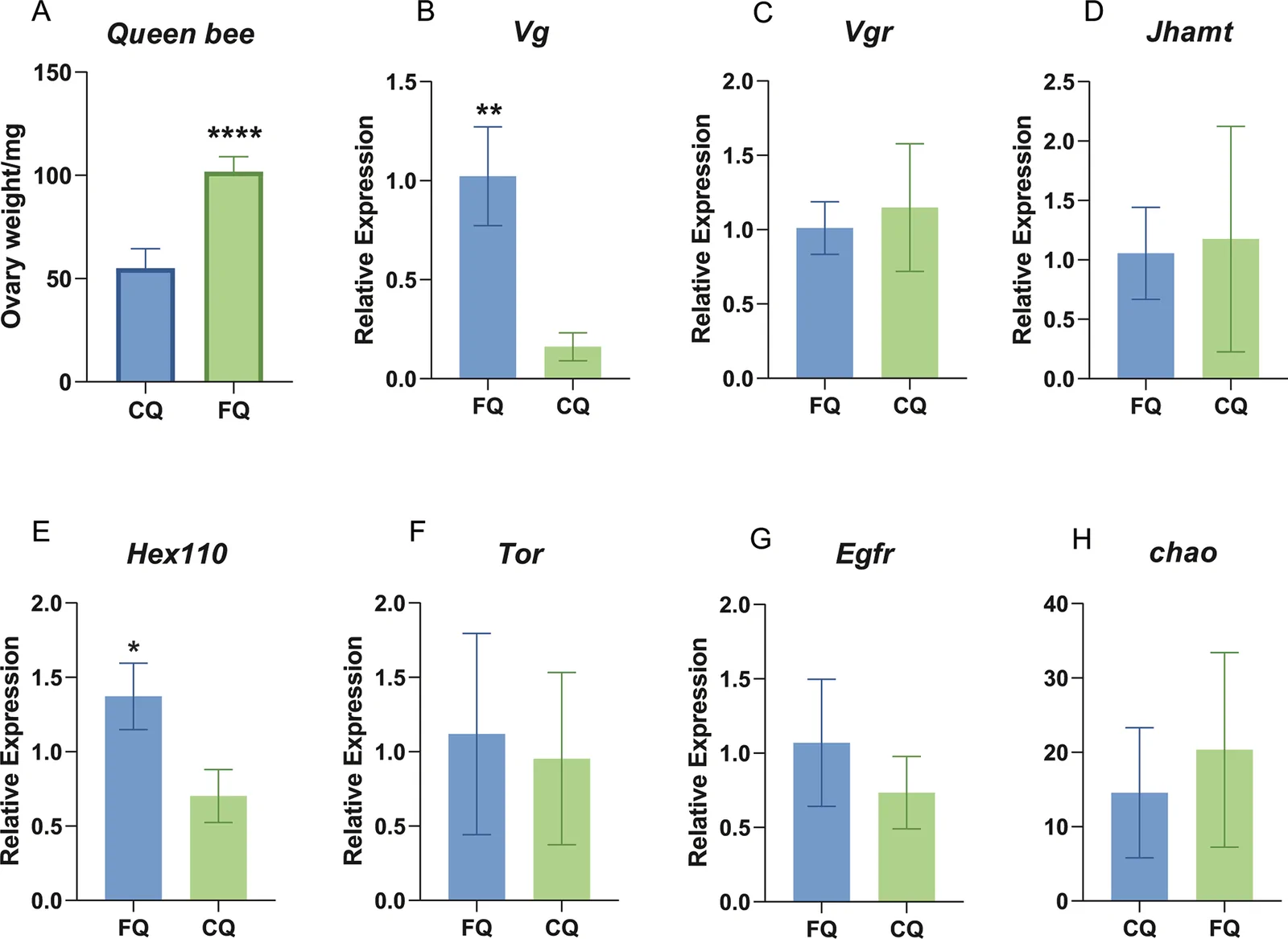
(**A**) Weight of queen bee ovaries. (**B–G**) Gene expression in the ovaries of queen bees. (**H**) Chao indices of alpha diversity in the queen bee gut. *P < 0.05, **P < 0.01, ****, *P* < 0.0001.

Fig. 1A shows the changes in queen ovary weight after 21 days of oviposition restriction. The results show that the weight of the queen ovaries changed significantly after 21 days of oviposition restriction, and the weight of the ovaries of the restricted queen (CQ) was almost half that of the unrestricted queen (FQ) compared to the unrestricted queen (*P* < 0.001).

After restricting the oviposition, there was a significant reduction in the expression of the *Vg* gene in queen ovaries (Fig. 1B). However, the relative expression of *Vgr* did not show any difference between the two groups (Fig. 1C). Additionally, the relative expression of *Jhamt*, a gene related to queen hierarchical differentiation, did not show any difference either (Fig. 1D). On the other hand, the expression of *Hex110*, a gene related to queen ovary development, was significantly higher in FQ ovaries compared to CQ ovaries (Fig. 1E). The expression of two nutrition-related genes, *Tor* and *Egfr*, did not differ between the two groups, but their expression was higher in FQ ovaries compared to CQ ovaries (Fig. 1F and G).

The results indicate that the development of the queen’s ovaries may be impeded and even regressed when the queen is restricted from laying eggs. However, it appears that the nutritional stress and access to food for the queen may not be as severe as initially anticipated.

### Changes in the gut flora of the queen bee

**FIG 2:**
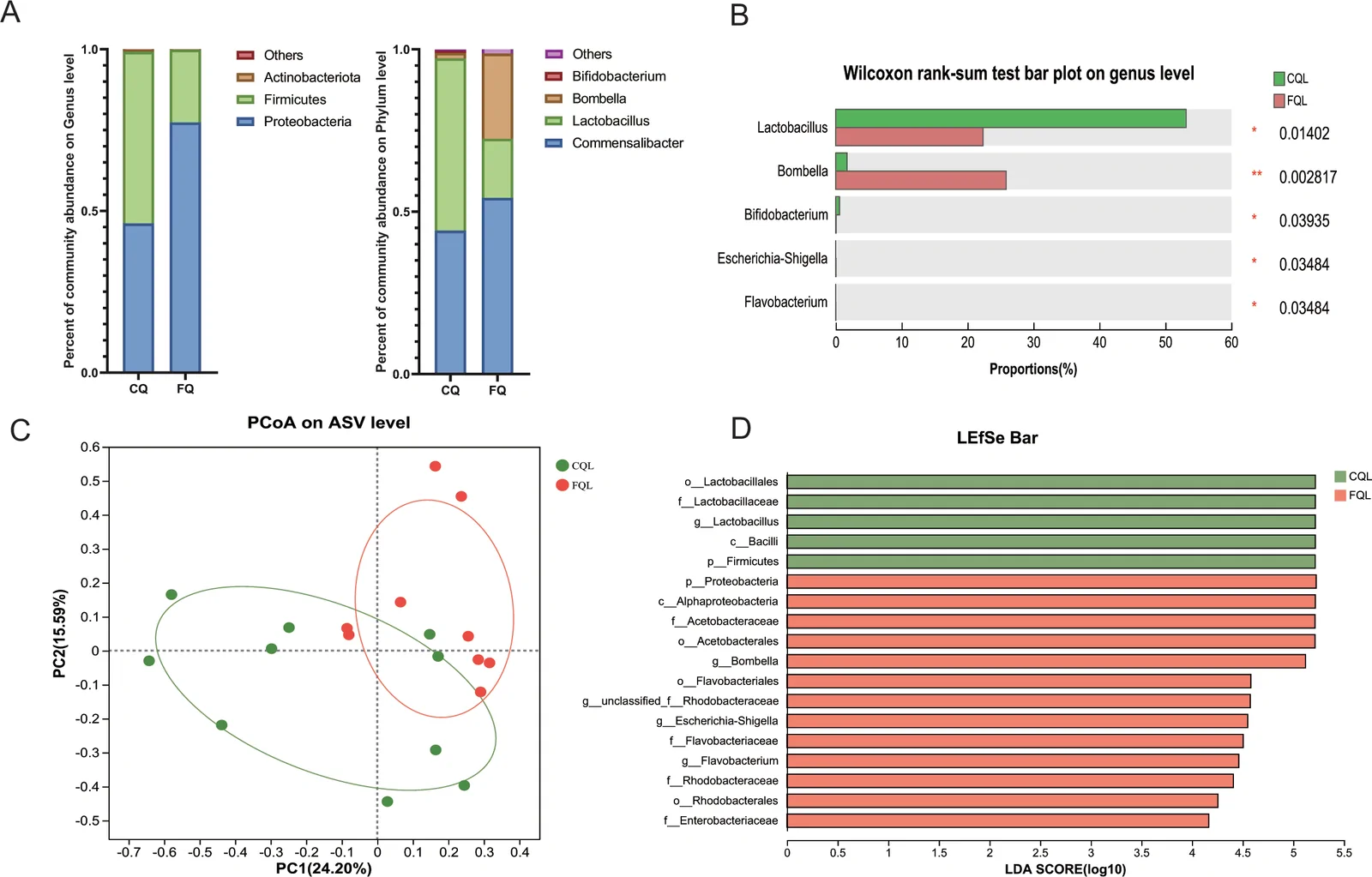
(**A**) The community distribution map of the queen bee’s gut microbiota at the phylum and genus levels. (**B**) At the genus level, the Wilcoxon rank-sum test was used to detect the differentially abundant genera in the gut microbiota of queen bees based on the abundance data of each genus in the samples. (**C**) PCoA of the queen bee gut microbiota at the ASV level. (**D**) LDA discriminant bar chart to summarize the microbial taxa with significant effects in each group of queen gut microbiota. *P < 0.05, **P < 0.01. LDA, linear discriminant analysis; LEfSe, linear discriminant analysis effect size; PCoA, principal coordinate analysis.

The richness of the gut microbiota in queen bees was reflected by the Chao index. Independent sample *t*-test analysis showed no significant difference in the Chao index between the two groups. However, compared to the CQ group, the FQ group exhibited higher gut microbiota richness. This result indicates that egg-laying restriction affects the abundance of gut microbiota in queen bees (Fig. 1H). At the phylum and genus level, oviposition restriction significantly altered the queen gut flora. The core phylum of the CQ gut flora changed from Proteobacteria to Firmicutes after queen oviposition restriction; the dominant flora of the gut flora also changed from *Commensalibacter* to *Lactobacillus*, and the diversity of the CQ gut flora decreased (Fig. 2A). The core of the microbiota changed significantly; the relative abundance of *Lactobacillus* increased from 22.34% to 53.14% (*P* = 0.03); the relative abundance of *Bombella* decreased from 25.85% to 1.72% (*P* = 0.008); the relative abundance of *Bifidobacterium* increased from 0.053% to 0.580% (*P* = 0.04); and the relative abundance of *Commensalibacter* did not change significantly (Fig. 2B).

Principal coordinate analysis (PCoA) is a non-restricted data dimensionality reduction method used to study the similarity or dissimilarity in the composition of sample communities. We employed PCoA to analyze the gut microbiota of two groups of queen bees. The results revealed that egg-laying restriction altered the composition and distribution of the gut microbiota in queen bees. There was a clear trend of separation between the gut microbiota of CQ and FQ groups, with a decrease in gut microbiota richness observed in the CQ group after egg-laying restriction (Fig. 2C).

Furthermore, a linear discriminant analysis effect size analysis was conducted to identify differential gut flora between the two groups (Fig. 2D). The findings revealed that *Lactobacillus* was more abundant in CQ, while *Bombella* was more abundant in FQ and significantly differed from the other group. Additionally, the diversity of gut bacteria was found to be greater in FQ compared to CQ. These results suggest that the queen’s gut flora diversity may decrease after oviposition restriction and that the queen’s core flora may undergo changes as a defensive mechanism against external environmental effects.

### Metabolomic analysis and metabolite identification

**FIG 3:**
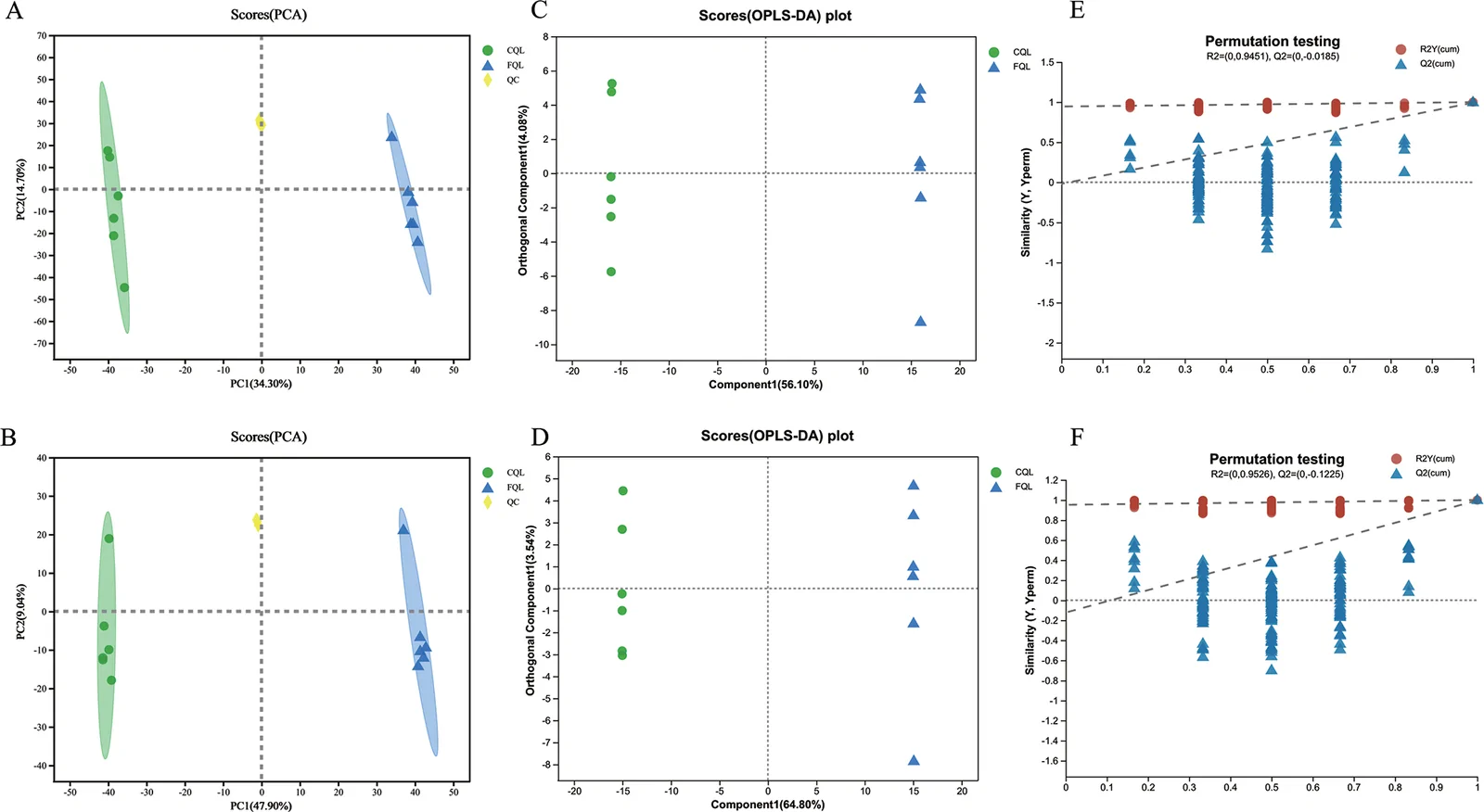
(**A and B**) The PCA score plots are shown separately in positive and negative ion modes. (**C and D**) The OPLS-DA score plots are shown in positive and negative ion modes, respectively. (**E and F**) The OPLS-DA permutation test plots are shown in positive and negative ion modes, respectively. OPLS-DA, orthogonal partial least squares discriminant analysis; PCA, principal component analysis.

Ultra-high performance liquid chromatography tandem mass spectrometry (UHPLC-MS) was used to obtain ovarian metabolite information of CQ and FQ. After processing, a total of 841 metabolites were identified in the two sample sets, including coenzyme factors, amino acids, nucleic acids, lipids, hormones, conducting substances, and carbohydrates (Table S2). Based on this metabolite information, an unsupervised model, principal component analysis (PCA), was constructed. The PCA score plot showed a clear separation trend between CQ and FQ in both the anionic and cationic modes, indicating that the two groups of queen bees differed in the composition of ovarian metabolites (Fig. 3A and B).

To further identify these metabolites, orthogonal partial least squares discriminant analysis (OPLS-DA) was performed. The OPLS-DA score plot was orthogonally rotated to filter out irrelevant information and allow better differentiation between the groups. The OPLS-DA score plot showed clear separation between CQ and FQ in both anionic and cationic modes (Fig. 3C and D). In addition, to avoid overfitting, the OPLS-DA results were evaluated using the OPLS-DA substitution test, with an R2 of 0.9451 in the cationic mode and 0.9526 in the anionic mode (Fig. 3E and F). These results suggest that the OPLS-DA model is reliable in both the cationic and anionic modes and can be used for further analysis of differential metabolites.

### Differential metabolite identification

These differential metabolites were selected based on a combination of a statistically significant threshold of variable influence on projection (VIP) values obtained from the OPLS-DA model and *P* values from a two-tailed Student *t*-test on normalized peak areas. Metabolites with VIP values greater than 1.0 and *P* values less than 0.05 were considered statistically significant.

**FIG 4:**
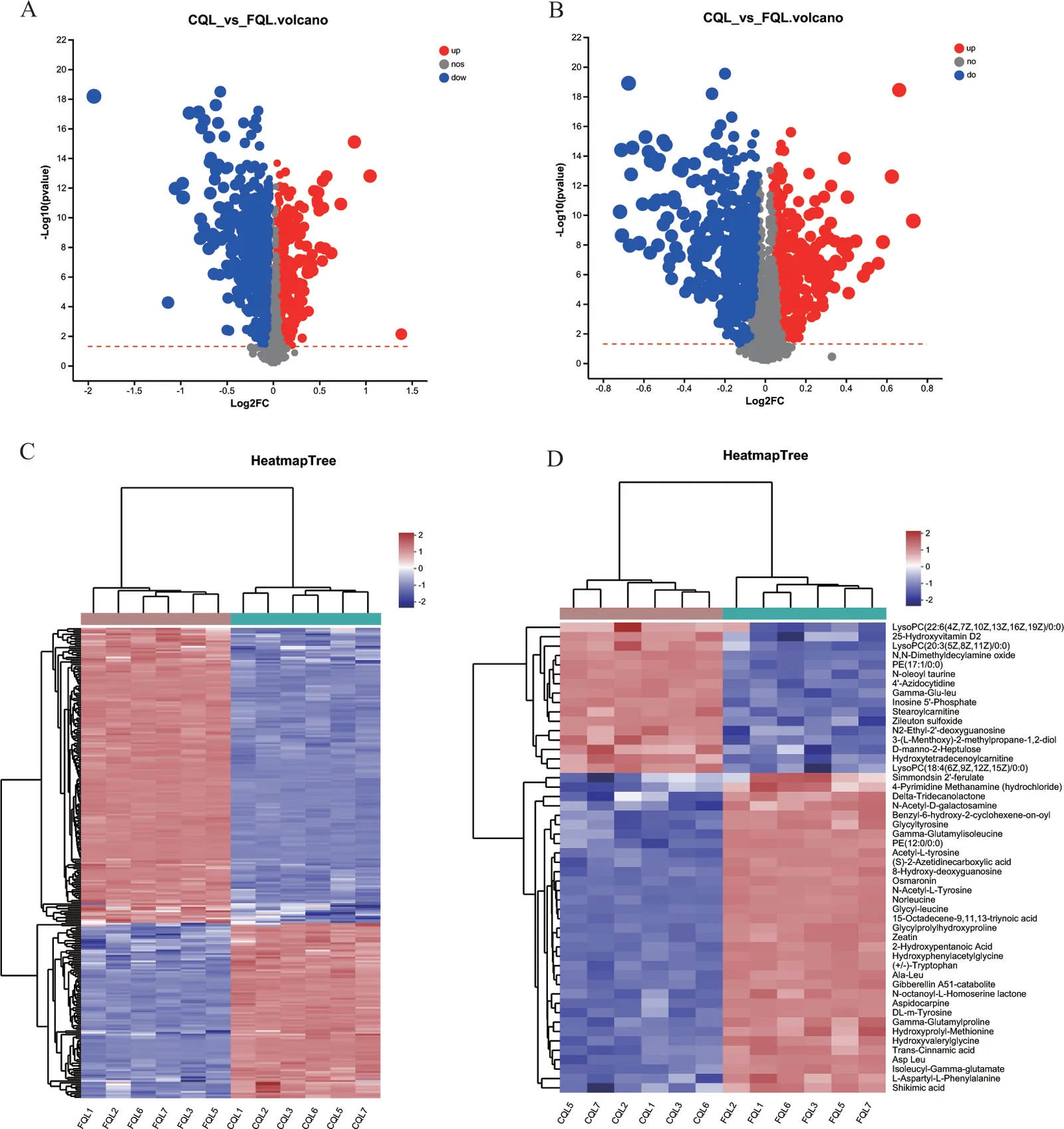
(**A and B**) The volcano plots are shown separately in positive and negative ion mode. (**C**) Cluster analysis of each sample with all metabolites, where red represents positive correlation and blue represents negative correlation. (**D**) The clustering analysis of each sample with the top 50 abundant metabolites, with red indicating positive correlation and blue indicating negative correlation.

Based on these differential metabolites, volcano plots were constructed to specifically display the metabolites within the two groups. In the cationic mode volcano plot, a total of 877 differential ion peaks were detected, with only 157 metabolites identified (Fig. 4A). In the anionic mode volcano plot, a total of 860 differential ion peaks were detected, with only 106 metabolites identified (Fig. 4B). The volcano plot revealed that the fold change values of metabolite expression differences between the two groups were higher in the cationic mode than in the anionic mode, but the statistical test values of the differences in metabolite expression changes were not significantly different between the two groups. A total of 263 named metabolites were detected (Table S3), of which 165 were upregulated and 98 were downregulated.

A clustering heat map was constructed based on these significantly different metabolites, and the distribution of each metabolite in the two groups was visualized in the heat map (Fig. 4C). The clustering analysis showed that restricting queen oviposition caused changes in metabolites in CQ ovaries, with a clear separation of metabolites between CQ and FQ, and that restricting queen oviposition caused a decrease in the expression of most metabolites in CQ ovaries and an increase in the expression of only a small fraction of metabolites. To better understand the changes in ovarian metabolites, we selected the 50 most abundant metabolites to construct a clustering heat map (Fig. 4D). The results showed that the expression of 34 metabolites was downregulated, and only 16 metabolites were upregulated compared to the control, which was similar to the results of the previous clustering heat map. This suggests that when the queen ovary is in a closed state, the ovary itself decreases some metabolic activities to adapt to this change.

### Metabolic pathway analysis

**FIG 5:**
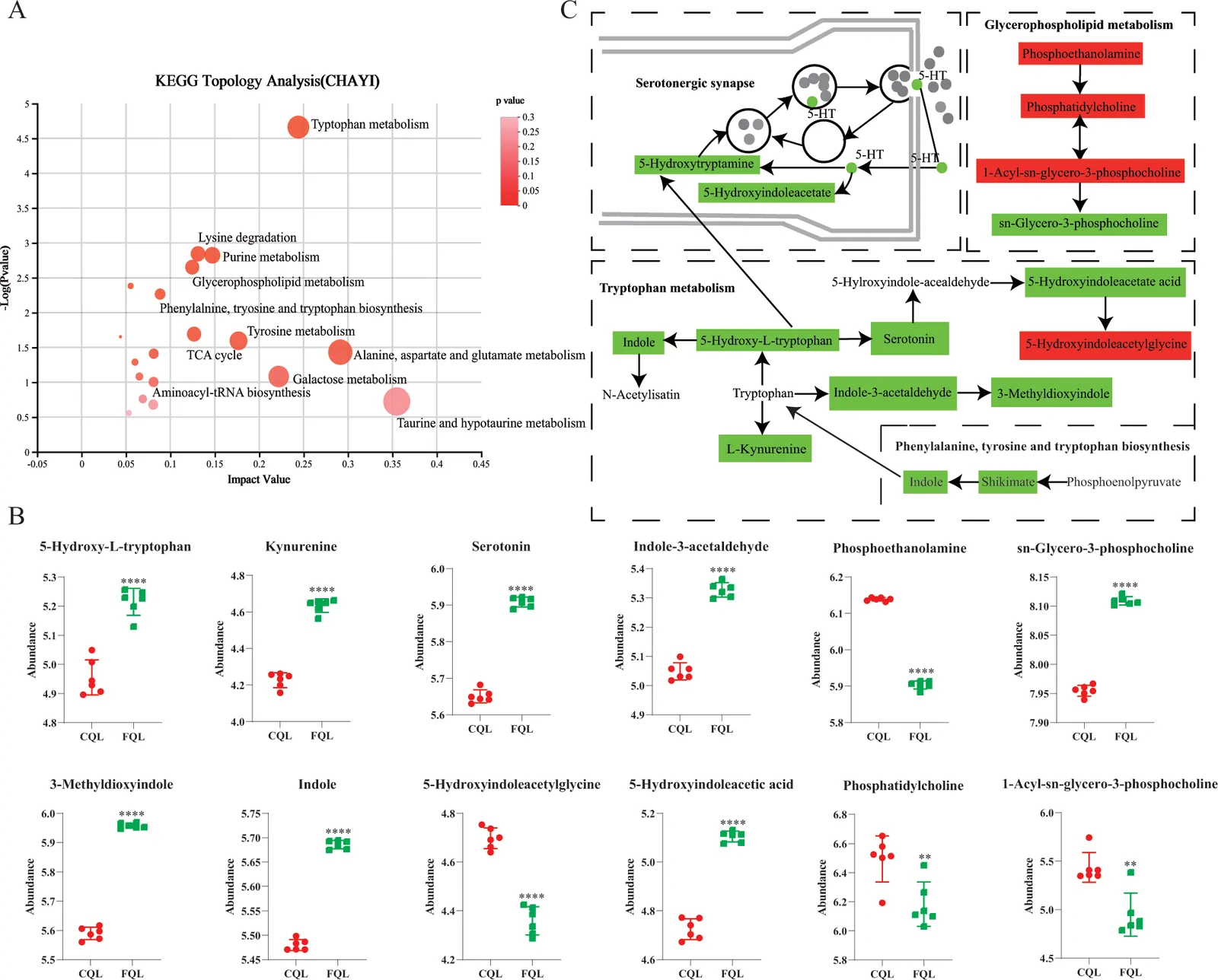
(**A**) Bubble plot of Kyoto Encyclopedia of Genes and Genomes (KEGG) topological analysis, where the size of the bubble indicates the importance of the pathway. (**B**) The changes of differential metabolites identified in phenylalanine, tyrosine, and tryptophan biosynthesis, Tryptophan metabolism, serotonergic synapse, and glycerophospholipid metabolism are shown with a red background indicating metabolites at higher levels, and a green background indicating metabolites at lower levels. (**C**) The specific expression of differential metabolites in phenylalanine, tyrosine, and tryptophan biosynthesis, tryptophan metabolism, serotonergic synapse, and glycerophospholipid metabolism. **P < 0.01, ****P < 0.0001. TCA, tricarboxylic acid cycle; CHAYI, the name of the metabolome set.

To further understand the changes occurring in the ovary, pathway enrichment analysis of differential metabolites was performed, and 79 pathways were enriched (Table S4), including tryptophan metabolism, glycerophospholipid metabolism, purine metabolism, and tyrosine metabolism (Fig. 5A), where tryptophan metabolism (*P* < 0.001) and glycerophospholipid metabolism (*P* < 0.01) were significantly affected.

In addition, specific changes in metabolites involved in tryptophan and glycerophospholipid metabolism were investigated using non-targeted metabolomics (Fig. 5B). In the tryptophan pathway, 5-hydroxy-L-tryptophan, serotonin, indole-3-acetaldehyde, 5-hydroxyindoleacetic acid, indole, 3-methyldioxyindole, and kynurenine metabolites were significantly lower (*P* < 0.001), while 5-hydroxyindoleacetylglycine was significantly higher (*P* < 0.001) in CQ ovaries after restriction of queen oviposition. However, in glycerophospholipid metabolism, metabolites such as phosphatidylcholine (PC) (*P* < 0.01), phosphoethanolamine (*P* < 0.001), and 1-acyl-sn-glycero-3-phosphocholine (*P* < 0.001) were significantly increased in the ovaries, whereas sn-glycero-3-phosphocholine was significantly decreased (*P* < 0.001).

Based on the complete metabolite ensemble of the KEGG database, the metabolic network of changes occurring in the ovary was mapped using metabolomics data, which can visualize the interactions between different metabolic pathways (Fig. 5C). The figure reveals that shikimate in the phenylalanine, tyrosine, and tryptophan biosynthesis pathway can promote the production of indole, which in turn stimulates the production of 5-hydroxy-L-tryptophan in the tryptophan pathway, leading to the production of 5-hydroxytryptamine (5-HT). The 5-HT can participate in various metabolic activities through serotonergic synapse, assisting the host in regulating metabolic activities to adapt to external changes. Moreover, the metabolic correlation network map can provide specific information about changes in the ovary’s metabolome, aiding in a better understanding of the queen’s changes and reducing risks for beekeepers and apiculture.

### Gut microbiota regulates queen bee ovarian metabolism

**Fig 6:**
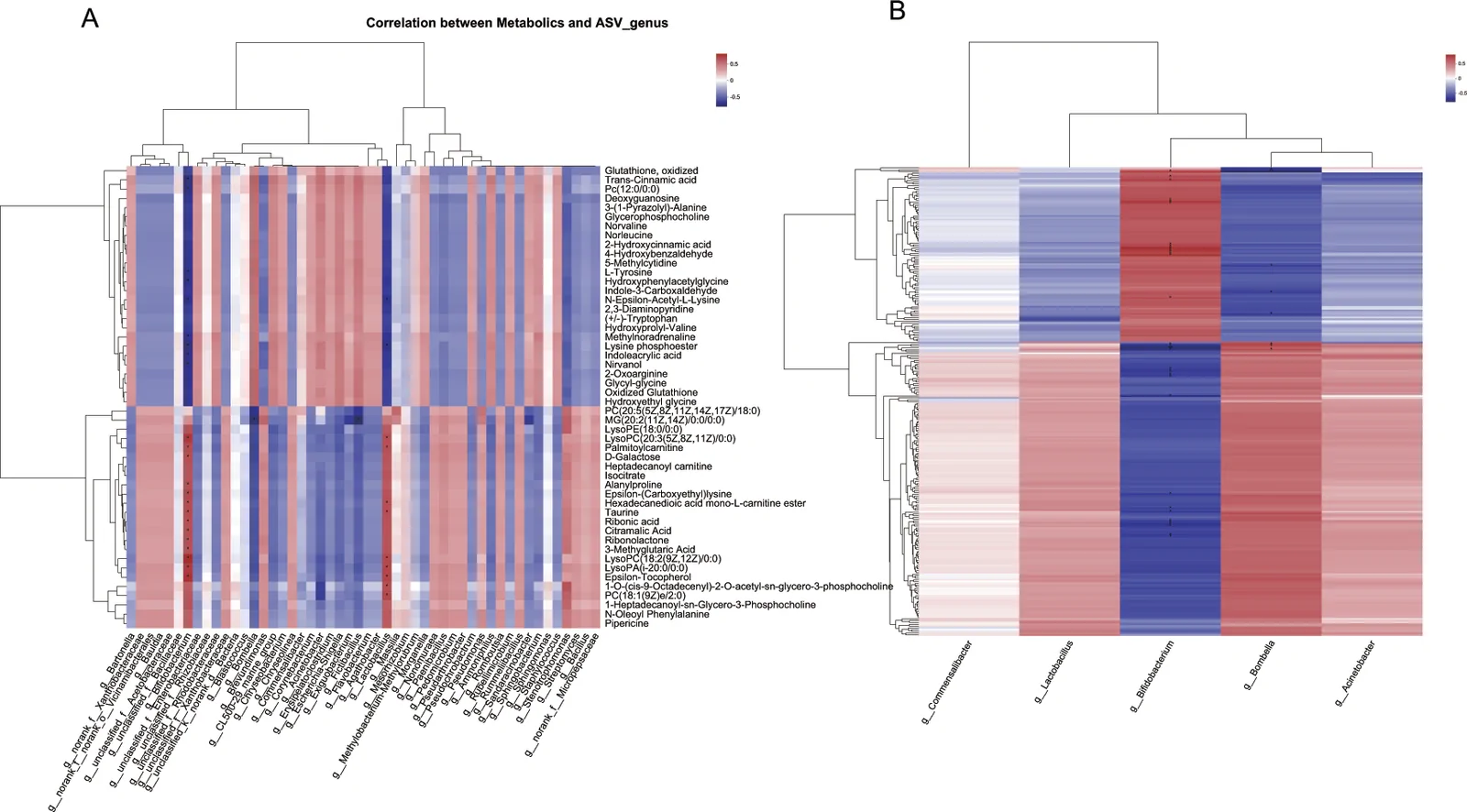
(**A**) Correlation analysis was performed between the top 50 metabolites and 16S rRNA data using Pearson’s correlation algorithm, and a heatmap was constructed using the top 50 associated features. (**B**) Correlation analysis was performed between the five core bacteria in the queen bee gut and all differential metabolites, and the darker the color, the greater the correlation coefficient.

To study the potential dependence between queen gut flora and ovarian metabolism, we used the Pearson correlation algorithm to calculate the correlation between the two data sets in volume and constructed a heat map of the proportional relationship between bacteria in the gut and the top 50 metabolites identified in abundance, and the top 50 association features in abundance were selected to form a correlation analysis-correlation heat map (Fig. 6A). The figure shows that there is a robust correlation between *Bifidobacterium*, *Bombella*, and *Lactobacillus* with numerous metabolites, while other gut bacteria exhibit partial involvement in modulating the metabolic activity of the queen bee’s ovary, although lacking a strong correlation with metabolites. These results imply that the gut microbiota of queen bees can influence the metabolic activity of the ovary, yet its impact appears to be relatively limited, with the ovaries themselves playing a more dominant regulatory role.

We selected *Commensalibacter*, *Lactobacillus*, *Bombella*, *Bifidobacterium*, and *Acinetobacter* from the queen’s gut flora to gain further insights into the relationship between the queen’s core gut bacteria and ovarian metabolism. The correlations between these five bacteria and 263 differential metabolites were calculated using the Pearson correlation algorithm, and a correlation heat map was constructed. The results revealed that the correlation between *Commensalibacter*, *Lactobacillus*, and *Acinetobacter* with ovarian metabolites was not particularly strong, while the correlation between *Bombella* and *Bifidobacterium* with ovarian metabolites was robust (Fig. 6B). This suggests that *Bombella* and *Bifidobacterium* may have the ability to regulate metabolites in the queen’s ovaries through metabolic pathways, helping the queen to safely navigate challenging periods and supporting the queen during egg laying due to environmental changes.

## DISCUSSION

In this study, we found that after restricting oviposition, the weight of CQ ovaries changed significantly, with the weight of the ovaries almost half of that of FQ. This is similar to previous reports that the weight of queen bee ovaries significantly decreased within 10 days after restricting oviposition (40). The longer the queen bee is restricted from laying eggs, the smaller the ovary becomes. When the queen bee is unable to lay eggs, the oocytes produced accumulate in the ovary, and the excess oocytes may in turn inhibit the rate of oocyte production. This leads to a reduction in the weight and size of the ovary of the queen bee (41).

A critical process in the maturation of most insect oocytes is the accumulation of *Vg* (42). Vitellogenin is synthesized primarily in the fat body of the honey bee abdomen and then transferred to the oocyte via the *Vgr* (43). When the queen bee is restricted from laying eggs, the number of oocyte cells in her ovaries may decrease, leading to a reduction in the amount of *Vg* in her ovaries. However, the expression level of *Vgr* remains constant and is not lowered due to oviposition restriction (40). In a closed state of the ovaries, the expression level of *Vgr* in the queen bee’s ovaries tends to remain stable to provide support when the queen bee begins to lay eggs due to changes in the environment.

*Jhamt* is an important enzyme involved in the biosynthesis of juvenile hormone (*JH*) in bees. *JH* is a known primary regulatory factor in the caste differentiation of bees (44). Previous studies have shown that applying a combination of sugar-rich royal jelly and *JH* to the larvae cells of queen bees is more likely to produce high-quality queen bees compared to larvae without this food supplement (45). Hexamerins would participate in the synthesis and utilization of amino acids during insect development. They may also function as JH-binding proteins. In addition, there is circumstantial evidence to support the hypothesis that larval hexamers are targeted for egg production (46, 47). *Hex110* is one of the four hexamerins in the honeybee. Due to the fact that *Hex110* is synthesized in large quantities by larval fat bodies and is widely secreted and stored in hemolymph, it meets the criteria for storage proteins in insects (48). However, there is also research showing that *Hex110* is located in the cytoplasm and nucleus of honey bee ovarian cells (49). Furthermore, the expression level of *Hex110* in the ovaries of mated egg-laying queen bees is higher than that in young virgin queen bees (50). Our results indicate that the expression of the *Jhamt* gene in the queen bee’s ovary does not change significantly after egg-laying restriction. Previous studies have shown that *Jhamt* is more abundant in queen bees than in worker bees (51). It is evident that the queen bee does not undergo caste differentiation due to the egg-laying restriction. Interestingly, after limiting oviposition, the expression level of *Hex110* in the queen’s ovary significantly decreased. When the queen stops laying eggs, the number of oocyte cells decreases, which may lead to a significant decrease in the expression of *Hex110* in the queen’s ovary. Oviposition not only causes changes in the behavior of the queen but also may affect differential gene expression.

In our study, when the queen bees were restricted from egg laying, they were also confined in a cage and only fed by worker bees that entered the cage. This resulted in increased nutritional stress for the queen bees (52). Gene expression of *Tor* and *Egfr*, which are related to nutritional stress, may be different under such conditions. However, our results showed that after egg-laying restriction, the expression of *Tor* and *Egfr* genes in the queen bee’s ovary only slightly decreased and did not significantly decrease. We believe that the expression of the *Tor* and *Egfr* genes may have significantly changed in the early stage of the queen bee’s restriction. However, after 21 days of restriction, the queen bees may have adjusted themselves through a series of metabolic activities, which led to no significant difference in the expression of *Tor* and *Egfr* genes between the two groups.

The gut microbiota of queen bees has rarely been studied. Our research found that the dominant bacteria in the gut microbiota of queen bees are *Commensalibacter*, *Lactobacillus*, and *Bombella*, and the diversity of the gut microbiota in queen bees is much lower compared to worker bees (33, 34). Interestingly, our study also revealed significant alterations in the gut microbiota of egg-limited queens. The gut microbiota of egg-limited queens exhibited striking similarity to that of aged queens. Specifically, there was a decrease in the abundance of Alpha 2.1 and other dominant bacteria in the gut of aged queens, while the relative abundance of core hindgut bacteria, such as *Lactobacillus* and *Bifidobacterium*, which are commonly considered probiotic, increased (33, 53). The highest relative abundance of bacteria in the CQ gut changed to *Lactobacillus*, which increased from 22.13% to 46.29%, and *Bombella*, which significantly decreased from 15.35% to 1.907%. The relative abundance of *Commensalibacter* did not change significantly, but it also decreased compared to FQ. This may be due to the fact that after the queen is restricted from laying eggs, the only source of food for the queen is the worker bees that enter the cage through the gaps in the cage to feed the queen (54). The queen has a single access to food, which can lead to a reduction in the diversity of the queen’s gut flora. In addition, the colonization of the queen’s gut flora is not completed in a few days as in the case of worker bees; it takes weeks or months to complete the colonization of the queen’s gut flora (55).

After egg-laying restriction, the gut microbiota of the queen bee changed, and we found that the gut microbiota of the queen bee with older age and lower productivity was extremely similar to that of the CQ (53). Previous studies have suggested that *Bombella* and *Commensalibacter* in the gut of queen bees may be related to their longevity (56), and both of these strains are part of the *Acetobacter* branch. There have been many reports on the reasons for the longevity of queen bees, but they all mention royal jelly. The significant difference between queen bees and worker bees is nutrition, and queen bees mainly eat royal jelly throughout their lives (57). The existing studies have shown that *Acetobacteriaceae* are abundant in the pharyngeal gland of nurse bees, royal jelly, and larvae fed with royal jelly, but can be negligible in the midgut and viscera of nurse bees and foraging bees (58). The queen bee’s gut is the digestive organ where royal jelly can enter, indicating that royal jelly may promote the proliferation of *Acetobacteriaceae* in the queen bee’s gut. Moreover, overwintering honey bee colonies with high abundance of *Acetobacteriaceae* show lower overwintering losses, indicating that *Acetobacteriaceae* plays a positive health role inside bees (59). Our results indicated that although there was no difference in the expression level of nutrition-related genes between the two groups, the expression level of nutrition-related genes in the queen bee did decrease after being subjected to egg-laying restriction. This suggests that the nutritional stress on the queen bee increased, which may lead to a decrease in the abundance of *Acetobacteraceae* in the queen bee gut, resulting in a decrease in queen bee lifespan and a decrease in the ability to adapt to changes in the external environment, causing irreversible damage to the bee colony. Moreover, after the queen bee restricts egg laying, the relative abundance of certain bacteria in the queen bee’s gut microbiota decreases, particularly a significant decline in *Bombella*. It is worth considering whether these bacteria are involved in the reproductive regulation of the queen bee.

The vitellogenin is mainly synthesized in the fat body and then transferred to the oocyte through the vitellogenin receptor (60). The queen’s ovaries utilize these lipid molecules to produce eggs, so the distribution of lipid molecules in the ovaries is crucial for the queen (61). Our results indicate that the glycerophospholipid metabolism pathway in CQ ovaries was significantly disturbed after limiting the queen’s oviposition. Glycerophospholipids are important lipid components in queen ovaries and play a crucial role in cell membrane structure and function. Previous studies have shown that glycerophospholipid metabolism in queen ovaries is closely related to their reproductive capacity (62).

The levels of phosphoethanolamine (PE), PC, and 1-acyl-sn-glycero-3-phosphocholine in the ovarian glycerophospholipid metabolism pathway were significantly increased in CQ compared to FQ. PE and PC are important components of biological membranes (63). Research suggests that PC in ovarian tissue prepares for egg production, promoting the maturation of oocytes and the development of the ovary (64). After restricting egg laying, some metabolic activities in the ovaries of queen bees begin to change, helping the queen to adapt to changes in the external environment. The levels of some metabolites in the glycerophospholipid metabolic pathway in the queen bee’s ovaries increase. These metabolites may promote the maturation of oocytes by regulating the composition and function of ovarian cell membranes, among other aspects. PC and PE accumulate during the growth process of oocytes, leading to an increase in the levels of these two metabolites.

The impact of tryptophan metabolism is most significant after restricting egg laying in queen bees. Many research results have shown that tryptophan metabolism plays an important role in animal ovaries; for example, tryptophan can promote ovarian development (65), possibly affecting hormone secretion (66) and promoting the maturation and development of ovarian follicles (67). Tryptophan is a precursor for serotonin (5-HT) synthesis, and tryptophan metabolism is an important mechanism for regulating serotonin. 5-HT has been detected in the oviduct, uterus, and ovary of various animals (including mice and hamsters) (68, 69). It has been shown that when 5-HT is reduced in the fallopian tubes and uterus of rats and this treatment is applied early in gestation, rats cannot produce neonates normally but can produce neonates when 5-HT levels return to normal (70). 5-HT can also induce ovulation, and serotonin injected directly into the gonads of clams can induce ovulation and enhance the fertilization capacity of sperm on oocytes (71). These results suggest that 5-HT is one of the important signaling molecules that regulate ovarian function, regulating ovarian development and function. 5-HT is able to regulate ovarian physiological functions, such as follicle development and maturation, by binding to receptors in the ovary, thus affecting egg production. This is similar to our findings; after the queen bee restricted her egg laying, the content of 5-hydroxy-L-tryptophan in the tryptophan metabolism pathway in the ovary decreased significantly, leading to a decrease in the content of 5-HT. 5-HT regulates the physiological activity of the queen bee through serotonergic synapses, adapting to changes in the environment. The metabolism of glycerophospholipids can maintain or store more lipid molecules, allowing the queen bee to quickly lay eggs when environmental conditions change. At this time, the queen bee will produce eggs with higher content of nutrients such as vitellogenin, which promotes egg development and leads to the formation of high-quality queen bees.

There is growing evidence that gut microbiota can provide essential amino acids and proteins to the host to maintain protein balance (72). Gut microbiota can also help the host resist external invasion by regulating the host’s immune response (73). *Bartonella* in worker bees can regulate the biosynthesis of amino acids in the bee body during winter, including several essential amino acids such as phenylalanine, tryptophan, and methionine. Providing essential amino acids in winter can help bees maintain their health (74). The core bacterium *Gilliamella* in bees can regulate the host’s carbohydrate metabolism and help the host obtain energy from it (75). *Lactobacillus* Firm4 and Firm5 can significantly alter the amino acid metabolic pathways of bees (76). *Bifidobacterium* acts as a core bacterium in the bee gut, helping the host digest polysaccharides (75). The research results indicate that the gut microbiota plays an important role in regulating the metabolic activity and immune response of honey bees. Different bacteria can provide essential amino acids, proteins, and energy for honey bees and also regulate the metabolic pathways inside the honey bees’ body to help them adapt to changes in the external environment. Our results suggest that *Bifidobacterium* and *Bombella*, which have a high correlation with differential metabolites in queen ovaries, may affect ovarian metabolic pathways by regulating some metabolic activities in the queen’s gut.

We believe that in the early stages of queen bee egg-laying restriction, *Bombella* (previously named *Parasaccharibacter apium* or Alpha 2.2) (77, 78) in the queen bee’s gut may help the queen bee adapt to environmental changes by regulating tryptophan metabolism in the queen bee’s ovaries (Fig. 5B). Once the queen bee has adapted to this change and her ovaries are always closed, *Bombella* in the queen’s gut may be further replaced by *Lactobacillus*, which becomes the dominant bacterium in the queen’s intestine. As mentioned earlier, when the queen bee is restricted from laying eggs, the relative abundance of *Bombella* in the CQ gut significantly decreases, and the relative abundance of *Lactobacillus* significantly increases. Moreover, we also found that the gut flora of older and less productive queens was very similar to that of the CQ gut flora. This result further supports the possibility that *Bombella* in the gut of queen bees may regulate ovarian development and function.

### Conclusion

In this study, we analyzed the gut microbiota and ovarian metabolites of queen bees. We found that when the queen bee’s egg laying was restricted, there was a decrease in gut microbiota diversity and a change in the core gut microbiota. Specifically, the relative abundance of *Bombella* decreased from 15.35% to 1.907%. This reduction weakened the queen’s resistance to environmental changes. From the perspective of ovarian metabolites, the queen’s ovaries exhibited decreased metabolic activity after egg-laying restriction, particularly in glycerophospholipid metabolism and tryptophan metabolism. Through integrated analysis, we discovered that *Bombella* in the queen bee’s gut microbiota may regulate the queen’s ovarian metabolism through tryptophan metabolism.

These findings provide new insights into the interaction between queen bee egg laying, gut microbiota, and ovarian metabolism. Further research can explore the precise role of *Bombella* and tryptophan metabolism in the physiological activities of queen bees, as well as better management and protection of bee colonies. Additionally, the methods used in this study can be applied to investigate the gut microbiota and metabolome of other insects or animals, providing new approaches for understanding the interactions between microbiota and hosts within organisms.

## Data Availability

Raw data for 16S rRNA of queen bee have been deposited under BioProject PRJNA949168 in the NCBI database. Metabolomic raw data have been uploaded to MetaboLights under the number MTBLS7576.

## SUPPLEMENTAL MATERIAL

Figure S1 spectrum.02145-23-s0001.docx Stacked bar graph of queen bee ovary weights.

Table S1 spectrum.02145-23-s0002.xlsx Primer sequences for real-time fluorescence quantitative PCR of queen bee ovaries.

Table S2 spectrum.02145-23-s0003.xlsx 841 metabolites in queen bee ovaries.

Table S3 spectrum.02145-23-s0004.xlsx 263 metabolites with names from queen bee ovaries.

Table S4 spectrum.02145-23-s0005.xlsx 79 metabolic pathways in the queen bee ovary.

ASM does not own the copyrights to Supplemental Material that may be linked to, or accessed through, an article. The authors have granted ASM a non-exclusive, world-wide license to publish the Supplemental Material files. Please contact the corresponding author directly for reuse.
