# Supplementary figures and images for: Gut microbiota-driven regulation of queen bee ovarian metabolism
Source: Microbiol Spectr. 2023 Sep 26;11(5):e02145-23. doi: 10.1128/spectrum.02145-23 (PMC10581225; doi:10.1128/spectrum.02145-23)

**Figure S1**. Stacked bar graph of queen bee ovary weights


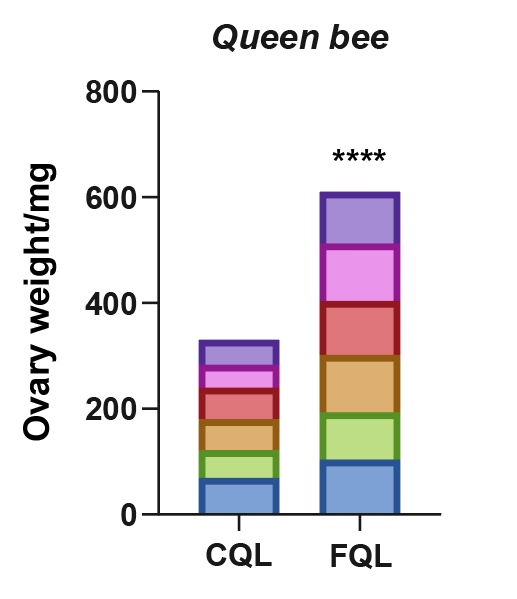

Supplement: Figure S1 — Stacked bar graph of queen bee ovary weights. [file spectrum.02145-23-s0001.docx]
